# ERF subfamily transcription factors and their function in plant responses to abiotic stresses

**DOI:** 10.3389/fpls.2022.1042084

**Published:** 2022-11-30

**Authors:** Ying Wu, Xiang Li, Jinnan Zhang, Haiqing Zhao, Shaolin Tan, Wanhao Xu, Jiaqi Pan, Fan Yang, Erxu Pi

**Affiliations:** College of Life and Environmental Sciences, Hangzhou Normal University, Hangzhou, China

**Keywords:** ERF subfamily, AP2/ERF superfamily, flavonoids, abiotic stresses, cis-elements

## Abstract

Ethylene Responsive Factor (ERF) subfamily comprise the largest number of proteins in the plant AP2/ERF superfamily, and have been most extensively studied on the biological functions. Members of this subfamily have been proven to regulate plant resistances to various abiotic stresses, such as drought, salinity, chilling and some other adversities. Under these stresses, ERFs are usually activated by mitogen-activated protein kinase induced phosphorylation or escape from ubiquitin-ligase enzymes, and then form complex with nucleic proteins before binding to *cis*-element in promoter regions of stress responsive genes. In this review, we will discuss the phylogenetic relationships among the ERF subfamily proteins, summarize molecular mechanism how the transcriptional activity of ERFs been regulated and how ERFs of different subgroup regulate the transcription of stress responsive genes, such as high-affinity K^+^ transporter gene *PalHKT1;2*, reactive oxygen species related genes *LcLTP*, *LcPrx*, and *LcRP*, flavonoids synthesis related genes *FtF3H* and *LhMYBSPLATTER*, etc. Though increasing researches demonstrate that ERFs are involved in various abiotic stresses, very few interact proteins and target genes of them have been comprehensively annotated. Hence, future research prospects are described on the mechanisms of how stress signals been transited to ERFs and how ERFs regulate the transcriptional expression of stress responsive genes.

## Introduction

Transcription factors (TFs) are able to interact with promoters of target genes, for activating or repressing their transcriptional expressions. As one of the largest transcription factor families in plants, the AP2/ERF (APETALA2/Ethylene Responsive Factor) plays indispensable roles in plant growth, development, hormone regulation, and especially in responses to various stresses (Tiwari et al., 2012; Lee et al., 2016; Xu et al., 2020; Yu et al., 2021). Commonly, members of the AP2/ERF family contain at least one copy of a DNA binding domain called the AP2 domain consisting of 60~70 amino acids (Liu et al., 2001; Magnani et al., 2004; Xie et al., 2019a; Zhang et al., 2020). The AP2 domain could be divided into two conservation acting elements, denominated as YRG and RAYD (Okamuro et al., 1997). The YRG element, consisting of about 20 amino acids, is a basic hydrophilic region for DNA binding. While the RAYD element, located at the C-terminus, is an amphiphilic region with the presence of an α-helix that can interact with other proteins or DNA (Owji et al., 2017; Ghorbani et al., 2020). Although the AP2 domain has been considered as plant specific, it was also found in the DNA binding domain of viral and bacterial HNH (His-Asn-His) endonuclease (A class of homing endonucleases) (Magnani et al., 2004). It is supposed that the gain of AP2 domain in plants was as a result of horizontal gene transfer *via* transposition and homing processes (Magnani et al., 2004).

Considering the variations in full-length of protein and in residue of AP2/ERF domain, members in this family have been categorized into series of subfamilies. Based on the sequence similarity of the Arabidopsis *(Arabidopsis thaliana)* annotated genome, Riechmann et al. (2000) firstly classified 144 Arabidopsis AP2/ERF members into three subfamilies, such as AP2, ERF, and RAV (Related to ABI3/VP1). Since not all ERF family members respond to ethylene induction, Sakuma et al. (2002) divided ERF proteins into two subfamilies, including the ethylene-related ERF subfamily and the ethylene-free DREB (Dehydration responsive element binding protein) subfamily. The DREB subfamily was further subdivided into six subgroups, called A-1 to A-6, while the six ERF subgroups were named as B-1 to B-6. Besides, a Soloist (few unclassified factors) subfamily was added into the AP2/ERF family (Sakuma et al., 2002). In this classification system, the AP2 subfamily contains two highly similar AP2 domains, while the ERF and DREB subfamilies each contains a single AP2 domain. Proteins of the DREB subfamily contain conserved amino acid residues at 14 (Val) and 19 (Glu) of the AP2/ERF domains, while those of ERF subfamily are Ala and Asp at position-14 and position-19 (Chen K. et al., 2022). In addition, the RAV subfamily contains an AP2 structure and a B3 structure, the Soloist subfamily contains an AP2 structural domain that differs significantly from those of other ERF transcription factors (Feng et al., 2020). Nakano et al. (2006) outlined the phylogenetic history of ERF transcription factors and groups functionally similar proteins together. They identified 147 AP2/ERF members in Arabidopsis and categorized them into four subfamilies, such as AP2, ERF, RAV, and Soloist. In which, the ERF and DREB subfamilies were combined as a novel ‘ERF subfamily’. In this study, all members of the abovementioned DREB and ERF subfamilies were included in the ERF subfamily. Based on the common amino acid sequence motifs outside of the AP2/ERF domain, the ERF subfamily was further divided into 12 groups, namely, groups I to X, VI-L (VI like) and Xb-L (Xb like) (Nakano et al., 2006; Zhu et al., 2021). To date, the classification scheme proposed by Nakano et al. (2006) is most extensively employed in literature.

The first ERF was identified as an ethylene response element binding protein from tobacco (*Nicotiana tabacum* L.) (Ohme-Takagi and Shinshi, 1995). With more extensive genome sequences, the identification and characterization of ERFs have been conducted in various plants. 122 ERFs have been found in *Arabidopsis thaliana* (Zhang et al., 2012), while there are 131, 104, 341, 166 and 323 homologues in *Oryza sativa* (Shao et al., 2020), *Triticum aestivum* (Zhuang et al., 2011), *Nicotiana tabacum* (Gao et al., 2020), *Zea mays* (Zhang J. et al., 2022) and *Glycine max* (Jiang et al., 2020), respectively. In addition, ERFs from different species were proven to regulate plant growth and development, immunity, and responses to various stresses (Muller and Munne-Bosch, 2015; An et al., 2020; Hong et al., 2022). Moreover, recent studies also showed that ERFs involved in the regulation of plant flavonoid synthesis (An et al., 2020; Zhao C. N. et al., 2021), which are essential to the homeostasis of ROS under abiotic stresses. However, it is still unclear how the variation of AP2/ERF domain in ERF subfamily affect their biological functions. In this study, phylogenetic analysis of the ERF subfamily in *Arabidopsis thaliana*, *Oryza sativa*, *Triticum aestivum*, *Nicotiana tabacum*, *Glycine max* and *Zea mays* will be conducted based on the sequence of the AP2/ERF domain, and the biological function of each subgroup will be specially discussed.

## Phylogenetic analysis

Whole-genome protein sequences of *Nicotiana tabacum*, *Arabidopsis thaliana*, *Glycine max*, *Oryza sativa*, *Triticum aestivum*, and *Zea may* were obtained from NCBI (https://www.ncbi.nlm.nih.gov/). The Hidden Markov model (HMM) profile of the AP2 domain (PF00847) was downloaded from the PFAM database (http://pfam.xfam.org/) (Zhang et al., 2021a), and was used to search candidate ERF proteins against the abovementioned whole-genome protein sequences of six species using the software HMMER3.0. After removal of redundant and incomplete sequences in CD-HIT, the putative ERFs sequences were analyzed with SMART (http://smart.embl.de/) and NCBI CD-search (https://www.ncbi.nlm.nih.gov/Structure/bwrpsb/bwrpsb.cgi) to confirm the presence and number of AP2 domain (Wang H. T. et al., 2022). Proteins that contain one AP2 domain but lack of B3 domain were retained as putative ERF proteins for phylogenetic analysis. A phylogenetic tree was subsequently constructed using the NJ method of MEGA 7.0, with bootstrap (1,000 replicates), and was visualized by the Interactive Tree of Life (iTOL). Finally, TBtools (Chen C. J. et al., 2020) was used to visualize the phylogenetic tree, conserved motifs and domain of AP2/ERF genes.

**Figure 1:**
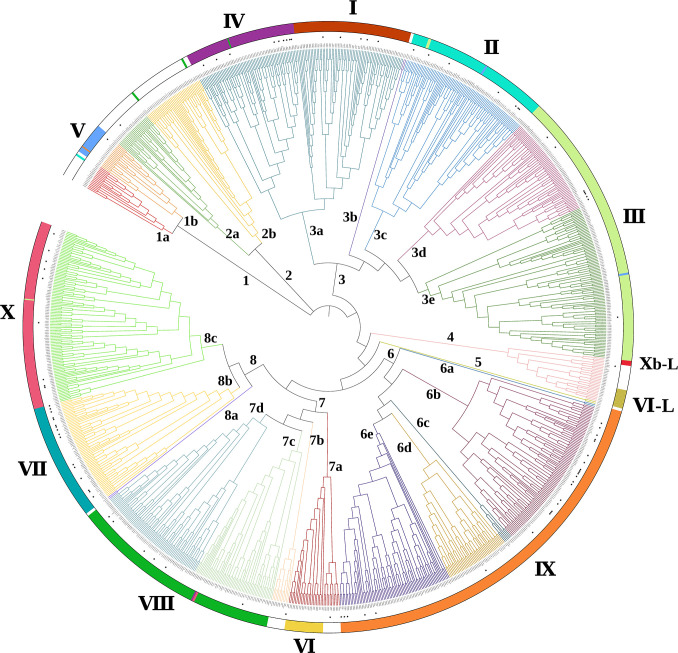
Phylogenetic relationships of ERF family members. The phylogenetic analysis of the ERF subfamily of *Nicotiana tabacum*, *Arabidopsis thaliana*, *Glycine max*, *Oryza sativa*, *Triticum aestivum*, and *Zea mays* by MEGA7.0 (Kumar et al., 2016), constructed the tree using the neighbor joining method and 1000 bootstrap replicates and used iTOL (https://itol.embl.de/) to visualize the tree (He et al., 2021; Magar et al., 2022). The category of functional annotated ERFs mentioned in this review which are listed in **Supplementary Table S2** are labeled with black star markers. ERF protein sequences were obtained from NCBI (https://www.ncbi.nlm.nih.gov) database. The protein ID and sequence of all these ERFs are listed in **Supplementary Tables S1**, **S3**. The ERF contained in each subgroup is present in **Supplemental Table S4**.

The phylogenetic distribution revealed that the 1117 ERFs were distributed into 8 groups which showed eight different clades labeled with Arabic numerals 1-8 and different line colors, where groups 1, 2, 3, 6, 7 and 8 could be further subdivided (**Figure 1**). Among the 23 subgroups, subgroup 3a has the largest number of members, consisting of 138 members. Whereas groups 3b, 5 and 8a contained only 2 members. The phylogenetic groups defined by Nakano et al. (2006) were designated as Roman numerals (I to X, VI-L, Xb-L) and were designated by 12 different colored circles outside. The white color means that the two categories do not correspond to each other. Comparing the two phylogenetic groups, we found that there are 12 ERF proteins with different classification compared to Nakano’s system, which is possibly due to conserved residues which might dominate biological functions of ERFs. For example, NP_196680.1 in subgroup IX according to Nakano was reassigned in subgroup 1b in the present phylogenetic tree. Proteins in the subgroup 1b, including the NP_196680.1, demonstrated with several conserved elements at specific positions, such as WLG, AYD, YRG and LNFP. Among them, WLG and YRG are supposed to play essential roles on biological functions of ERFs in 1b subgroup (Gao et al., 2020). However, these residues are not conserved in members of subgroup IX. Based on similar principle, NP_197901.1(II), NP_178173.1(VIII), NP_197480.1(VIII), NP_197346.1(VIII), NP_177301.1(III), NP_174636.1(III), NP_680184.1(V), NP_196720.1(V), NP_196895.1(X), NP_197357.2(IV), NP_196837.1(III) are reassigned to 1a, 2b, 2b, 3a, 3c, 3c, 3c, 3e, 7c, 7c and 8c, respectively.

## ERFs of different subgroup selectively bind to particular cis-acting elements of target genes

**Figure 2:**
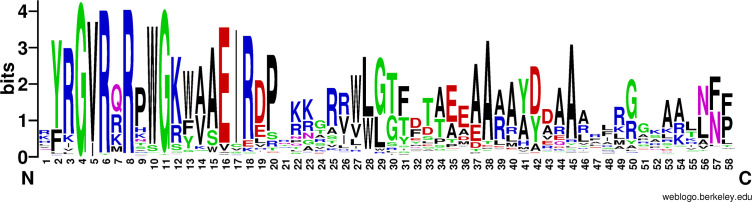
The motif-logo of DNA binding domains in ERF proteins. The motif-logo was plotted with multiple comparisons of 1117 AP2 domains using the software Weblogo (http://weblogo.berkeley.edu/logo.cgi). The overall height of the stack indicates the conservativeness of the sequence at that position, while the height of the letters within the stack indicates the relative frequency of each amino group at that position.

Commonly, there are some core residues conserved in DNA binding domain of transcription factors that reflect structural and functional specificities in a certain family. A sequence alignment was used to find the conserved residues in the ERF/AP2 DNA binding domain (Nakano et al., 2006; Xie et al., 2019b) of 1117 ERF proteins (**Figure 2** and **Supplementary Figure S1**). It was found that residues Tyr-2 (Y), Arg-3(R), Gly-4 (G), Val-5 (V), Arg-6 (R), Arg-8 (R), Arg-9 (R), Trp-10 (W), Gly-11 (G), Lys-12 (K), Trp-13 (W), Ala-14 (A), Ala-15 (A), Glu-16 (E), Ile-17 (I), Arg-18 (R), Asp-19 (D), Pro-20 (P), Arg-25 (R), Trp-27 (W), Leu-28 (L), Gly-29 (G), Glu-35 (E), Ala-37 (A), Ala-38 (A), Ala-40 (A), Asp-42 (D), Ala-44 (A), Ala-45 (A) and Gly-50 (G) are conserved among most (> 50%) ERF proteins. In addition, the residues Val-14 (V), Trp-28 (W), Leu-29 (L), Thr-33 (T), Ala-34 (A), Tyr-41 (Y) and Asn-57 (N) could also be found in about 40% ERF proteins. These conserved amino acids might determine their binding activity to different *cis* elements (Yamaguchi-Shinozaki and Shinozaki, 1994; Fujimoto et al., 2000). For example, the Val-14 (V) was previously proven as a key residue in the β-sheet of the ERF/AP2 domain for binding with DRE element (Zhuang et al., 2016), while Ala-37 (A) was predicted as a crucial residue of the α-helix in the DNA binding domain or as an important element for the stability of the ERF/AP2 domain (Liu et al., 2006).

Usually, members of the ERFs subfamily prefer to recognized the Ethylene-Response Element (ERE) with GCC-box sequence (AGCCGCC) which presents in the promoters of ethylene inducible pathogenesis-related (PR) genes as well as some genes associating abiotic stresses, while the DREBs subfamily recognizes Dehydration-Responsive or C-Repeat Element (DRE/CRT) with a core motif of [(A/G)CCGAC] in promoters of target genes to confer resistance to abiotic stresses, especially drought and cold (Yamaguchi-Shinozaki and Shinozaki, 1994; Ohme-Takagi and Shinshi, 1995; Stockinger et al., 1997; Zarei et al., 2011; Yang et al., 2018; Debbarma et al., 2019; Xie et al., 2019b; Zhang J. et al., 2022). These two primary *cis*-elements have been identified in several plant species, such as Arabidopsis (Arabidopsis thaliana) (Yang et al., 2009), rice (Oryza sativa) (Mohanty, 2021), wheat (*Triticum turgidumssp*) (Xing et al., 2017), soybean (*Glycine max*) (Jiang et al., 2020), tomato (*Lycopersicon esculentum*) (Yang H. H. et al., 2021), maize (*Zea mays*) (Hao et al., 2020) and tobacco (*Nicotiana tabacum*) (Gao et al., 2020).

The GCC-box is one of the most common targets in ERF subgroups 3a, 6b, 6e, 7a, 7d, 8b and 8c. Using heteronuclear multidimensional NMR techniques, Allen et al. (1998) reported the first three-dimensional solution structure of AP2/ERF domain in AtERF1 from Arabidopsis, and the secondary structure resembled the zinc finger which contains α-helices. It was found that the α-helix and β-sheet of AP2/ERF domain can recognize the core *cis*-acting elements GCC-box of the ethylene-responsive promoter region in target genes (Hao et al., 1998). Actually, the β-sheet in ERFs was more important for formation of the domain-GCC box complex (Sakuma et al., 2002). Rong et al. (2014) showed that the TaERF3 protein bound to the GCC-box *cis*-element in the promoters of seven stress-related genes, which positively regulated the responses to drought and salinity in wheat. Han et al. (2016) demonstrated that MaERF11 bound to the GCC-box motif of three ripening-related Expansin genes (*MaEXP2*, *MaEXP7* and *MaEXP8*), as well as an ethylene biosynthetic gene (*MaACO1*) in banana (*Musa acuminata*). Zhao et al. (2017) found that soybean GmERF113 bound to the GCC-box in pathogenesis-related (PR) genes, *PR1* and *PR10-1*, and positively regulated their expressions to increase resistance to *Phytophthora sojae* infection. Fang et al. (2022) further showed that the GmERF113 also positively regulated the drought response in soybean by activating *GmPR10-1* gene. In addition, Park H. C. et al. (2021) showed that AtERF72 was confirmed to recognize the GCC box in the promoters of several *PR* genes and activate their transcription, which could be enhanced by AtMPK6-induced phosphorylation. Zheng H. et al. (2021) confirmed that the interaction between SmERF73 and the GCC-box promoter elements of four tanshinone-associated genes regulated tanshinone biosynthesis in response to stress elicitors in *Salvia miltiorrhiza*. Besides, Zang et al. (2021) showed that the maize ZmERF061 may directly activate the expression of downstream defense-related genes by interacting with the GCC-box element in their promoter regions. In *Lilium longiflorum*, the heat-inducible LlERF110 may hinder the establishment of thermotolerance *via* being recruited to GCC-elements (Li T. et al., 2022). Zhu et al. (2022) suggested that VaERF16 from Chinese wild grape (*Vitis. amurensis* ‘Shuang You’) increased the transcript levels of *VaPDF1.2* by binding directly to the GCC box in its promoter, enhanced resistance of grapevine to *Botrytis cinerea* infection.

The DRE/CRT element could be recognized by ERFs which are mainly from subgroups 3a, 3d, 3e, 6b, 6e, 7a and 8b. The DRE element was identified from the promoter region of the *RD29A* gene which was involved in drought resistance in Arabidopsis (Yamaguchi-Shinozaki and Shinozaki, 1994; Zhao et al., 2013). And the CRT element was a similar motif with DRE element in cold-inducible genes (Baker et al., 1994; Zhao et al., 2013). Azzeme et al. (2017) showed that the elevated accumulation of *Elaeis guineensis* DREB1 in transgenic seedlings regulated the expressions of eight DRE-containing genes by interacting with DRE motif in their promoters under both oxidative and cold stress. Additionally, ZmDREB1A induces ABA-independent genes like *COR15A, KIN1*, and *KIN2* through binding their DRE/CRT sequences to further affect the expressions of dehydration and cold-responsive genes (Qin et al., 2004; Kimotho et al., 2019). Feng et al. (2019) found that zoysiagrass (*Zoysia japonica*) ZjDREB1.4 protein, which enhanced Arabidopsis tolerance to temperature stresses, was capable of binding specifically but weakly to the DRE/CRT element. Besides, Lee et al. (2021) demonstrated that the Arabidopsis CBF1 can bind to DRE/CRT motifs in the promoter of *COR15a* to confer freezing tolerance. Similarly, CBF2 and CBF3 of Arabidopsis, bind to the DRE, being also related to low-temperatures (Wu et al., 2017; Kidokoro et al., 2020). Mao et al. (2020) confirmed that SlDREBA4 specifically bound to the DRE elements of the downstream *Hsp* genes and contributed to heat tolerance in tomatoes (*Solanum lycopersicum*). However, recent study indicated that some DREB was also involved in certain processes during the plant life cycle *via* binding to DRE/CRT motifs. In transgenic cotton, Lu et al. (2022) suggested that not only did AmCBF1 from *Ammopiptanthus mongolicus* enhance cotton drought and cold stress tolerance, but it was capable of binding to the CRT/DRE elements in the upstream promoter of *GhPP2C1* or *GhPP2C2* and repressing their expression, which led to cotton dwarfing.

Despite the generalization shown previously that the ERFs bind to GCC-box and the DREBs bind to DRE/CRT element, an increasing number of studies have been reported that some ERFs are also capable of binding to DRE/CRT elements and vice versa. (Sun et al., 2008), implying their potential roles in abiotic stress (Eini et al., 2013; Thirugnanasambantham et al., 2015; Behera et al., 2022). For instance, Tsi1, an ERF protein from tobacco, binds specifically to the GCC and the DRE/CRT sequences, resulting in improved tolerance to salt and pathogens (Park et al., 2001). Zhang G. Y. et al. (2009) suggested that GmERF3 specifically bound to both the GCC box and DRE/CRT element to enhance the soybean’s tolerances to salinity, drought as well as pathogen infection. CaPF1, a pepper (*Capsicum annuum*) ERF, was proved to confer pathogen and freezing tolerance in transgenic Arabidopsis through binding to GCC box and DRE/CRT element (Yi et al., 2004). TSRF1, an ERF protein from tomato, binds to both GCC-box and DRE sequences to promote drought and osmotic tolerance in some transgenic plants (Zhang et al., 2004; Quan et al., 2010). In addition, LcERF056 from *Lotus corniculatus* bound to *cis*-element GCC-box or DRE of reactive oxygen species (ROS)-related genes to enhance plant salt tolerance (Wang D. et al., 2021). By binding to the GCC and DRE *cis*-elements, OsERF096 activated the expression of unknown targets to regulate cold tolerance of rice *via* JA-mediated signaling (Sun et al., 2022). Chen N. N. et al. (2022) indicated that PalERF2 regulated drought response in poplar (*Populus alba* var. *pyramidalis*) by binding to DRE motifs on the promoters of drought-responsive genes *PalRD20* and *PalSAG113*. In walnut (*Juglans regia*), Yang G. Y. et al. (2021) revealed that JrERF2-2 effectively improved plant drought tolerance through interacting with JrWRKY7 to control the expression of *GSTs* by binding to GCC-box or DRE motif. Finally, IbRAP2.4 from *Ipomoea batatas* bound to both DRE and GCC-box elements, which promoted lateral root formation and enhanced the drought tolerance of transgenic Arabidopsis, while it inhibited storage root formation in transgenic sweet potato by comprehensively up-regulating lignin biosynthesis pathway genes (Bian et al., 2022).

In particular, some ERF and DREB members bound to both abovementioned elements in promoter of genes involved in hormone pathway, like ethylene, jasmonic acid and auxin. For instance, the tomato (*Solanum lycopersicum* [f. sp. *Lycopersicon esculentum*]) LeERF2 and DRE could form as a transcriptional complex on the promoter and activate the expression of *LeACO3* for ethylene biosynthesis (Zhang Z. J. et al., 2009). In addition, Li et al. (2016) found that Apple (*Malus domestica*) MdERF2 interacted with the DRE motif of *MdACS1* gene and suppressed its transcription, thereby inhibiting ethylene biosynthesis in ripening fruit. In Chinese flowering cabbage (*Brassica rapa* var. *parachinensis*), Tan et al. (2018) revealed that BrERF72 directly activated expressions of JA biosynthetic genes *BrLOX4, BrAOC3*, and *BrOPR3* through binding to GCC or DRE/CRT *cis*-element during JA-promoted leaf senescence. Besides, Liu et al. (2018) demonstrated that tomato Sl-ERF.B3 could regulate the expression of *Sl-IAA27* probably through directly binding to the typical DRE/CRT element presenting in its promoter region, while *Sl-ERF.B3* was associated with ethylene and auxin signaling. The reason for their different DNA-binding specificity depends on the divergent residues in β-sheet of AP2/ERF domain (Thirugnanasambantham et al., 2015). In which, some residues dominate the binding ability of ERFs and DREBs to DRE/CRT and/or GCC-box. For example, the Val-14 and Glu-19, especially Val-14, were proven to be essential for specific binding to DRE (Sakuma et al., 2002). In addition, Liu et al. (2006) indicated that Ala-37 was reported to play a key role in binding to both GCC-box and DRE, while Sun et al. (2008) shown that the Ser-15 in the AP2/ERF domain was demonstrated to be essential for its specific binding to GCC-box. Recent studies showed that some other amino acids, such as Pro-9, His-9 and Ser-9, also have important functions (Liu et al., 2020; Zhang L. et al., 2022).

Additionally, ERFs can also recognize other *cis*-elements that diverge significantly from the abovementioned two motifs (Welsch et al., 2007; Shaikhali et al., 2008; Xie et al., 2019b). For example, ERF75/RAP2.2 regulated carotenoid biosynthesis pathway in Arabidopsis *via* directly binding to 5′-ATCTA-3′ sequences of genes *PSY* and *PDS* (Welsch et al., 2007). Besides, RAP2.2 also bound to hypoxia-responsive promoter elements (HRPE) (5′-AAACCA(G/C)(G/C)(G/C)GC-3′) to regulate Hypoxia-Responsive gene expression in Arabidopsis (Gasch et al., 2016). In addition, RRTF1/ERF109 participated in ROS homeostasis under dehydration stress through binding to GCC box-like motif (AGACGCC) of genes *ZAT12* (Wang et al., 2020).

In short, ERFs recognize and bind to various *cis*-elements which in the promoters of target genes to participate in different regulatory processes by regulating their expression (**Table 1**). We found that recent researches on *cis*-elements of ERF mainly focused on 4 species, like *Arabidopsis thaliana*, *Glycine max*, *Solanum lycopersicum* and *Zea mays*. Moreover, most of ERFs positively regulated the expression of target genes through binding to their *cis*-elements, only one tenth of ERFs play negative regulatory roles. Finally, ERFs of different subgroups were inclined to recognize different *cis*-elements. It is suggested that the ERF members in subgroups 3a, 6b, 6e, 7a and 8b are capable of recognizing cis-elements in GCC-box and/or DRE/CRT element. The subgroups 7d and 8c are more likely to bind to GCC-box, while ERFs in subgroup 3d and 3e have more possibilities to recognize DRE/CRT element.

**Table 1:** Mechanism of ERF on transcriptional regulation of target genes.

*cis*-acting element	Plant species	Nomenclature	Target gene	Regulation of target gene	Regulation of biological response	Subgroup	Effect	References
DRE/CRT	*Ammopiptanthus mongolicus*	AmCBF1	*GhPP2C1, GhPP2C2*	Negative	Positive	3d	Regulate plant dwarf phenotype	Lu et al., 2022
GCC box	*Arabidopsis thaliana*	AtERF72	unknow	Positive	Positive	8b	Participate in plant resistance to pathogenesis	Park H. C. et al., 2021
GCC box	*Brassica rapa* var. *parachinensis*	BrERF72	*BrOPR3*	Positive	Positive	8b	Regulate JA-promoted leaf senescence	Tan et al., 2018
DRE/CRT			*BrLOX4, BrAOC3*	Positive	Positive		Regulate JA-promoted leaf senescence	Tan et al., 2018
GCC box	*Capsicum annuum*	CaPF1	*PDF 1.2*	Positive	Positive	8b	Regulate pathogen infection; paticipate in freezing tolerance	Yi et al., 2004
DRE/CRT			*OR47, COR6.6, COR78/RD29*	Positive	Positive		Regulate pathogen infection; paticipate in freezing tolerance	Yi et al., 2004
DRE/CRT	*Arabidopsis thaliana*	CBF1	unknow	Positive	Positive	3d	Participate in freezing tolerance	Lee et al., 2021
DRE/CRT	*Arabidopsis thaliana*	CBF2	*AtCOR, AtCOR15A, AtKIN1, AtRD29A, AtSuSy*	Positive	Positive	3d	Participate in low-temperatures tolerance	Wu et al., 2017; Kidokoro et al., 2020
DRE/CRT	*Arabidopsis thaliana*	CBF3	unknow	Positive	Positive	3d	Participate in low-temperatures tolerance	Wu et al., 2017; Kidokoro et al., 2020
DRE/CRT	*Elaeis guineensis*	EgDREB1	*LePOD, LeAPX, LeGP, LeCAT, LeHSP70, LeLEA, LeMET2, LePCS*	Positive	Positive	3d	Participate in oxidative and cold tolerance	Azzeme et al., 2017
HRPE	*Arabidopsis thaliana*	ERF75/RAP2.2	*LBD41, PCO1*	/	/	8b	Regulate limitied oxygen	Gasch et al., 2016
ATCTA			*PSY, PDS*	/	/		Regulate carotenoid pathway	Welsch et al., 2007
GCC box	*Glycine max*	GmERF113	*PR1, GmPR10-1*	Positive	Positive	8c	Participate in resistance to Phytophthora sojae infection; participate in drought tolerance	Zhao et al., 2017; Fang et al., 2022
GCC box	*Glycine max*	GmERF3	*PR1, PR2, PR4, Osmotin, SAR8.2*	Positive	Positive	8b	Regulate pathogen infection	Zhang G. Y. et al., 2009
DRE/CRT			unknow	/	Positive		Participate in salt or drought tolerance	Zhang G. Y. et al., 2009
DRE,GCC box	*Ipomoea batatas*	IbRAP2.4	*PAL, C4H, CAD, CCR, COMT, CCoAOMT*	Positive	Positive	3a	Regulate storage root formation and lignin biosynthesis	Bian et al., 2022
GCC box	*Juglans regia*	JrERF2-2	*JrGST4, JrGST6, JrGST7, JrGST8, JrGSTF8*	Positive	Positive	6b	Participate in cold tolerance	Yang G. Y. et al. (2021)
DRE/CRT			*JrGST11, JrGST12, JrGSTN2*	Positive	Positive		Participate in cold tolerance	Yang G. Y. et al. (2021)
GCC box	*Lotus corniculatus*	LcERF056	*LcPrx, LcRP*	Positive	Positive	6b	Participate in salt tolerance	Wang D. et al., 2021
DRE/CRT			*LcLTP*	Positive	Positive		Participate in salt tolerance	Wang D. et al., 2021
GCC box	*Solanum lycopersicum*	LeERF2	*NtACS3*	Positive	Positive	8b	Regulate ethylene biosynthesis	Zhang G. Y. et al. (2009)
DRE/CRT			*LeACO3*	Positive	Positive		Regulate ethylene biosynthesis	Zhang G. Y. et al. (2009)
GCC box	*Lilium longiflorum*	LlERF110	unknow	/	Negative	8c	Participate in heat tolerance	Li T. et al., 2022
GCC box	*Musa acuminata*	MaERF11	*MaACO1, MaEXP2, MaEXP7, MaEXP8*	Negative	Negative	7d	Regulate fruit ripening; Regulate ethylene biosynthesis	Han et al., 2016
DRE/CRT	*Malus domestica*	MdERF2	*MdACS1*	Negative	Negative	6b	Regulate ethylene biosynthesis	Li et al., 2016
DRE,;GCC box	*Oryza sativa*	OsERF096	unknow	Positive	Positive	6b	Participate in cold tolerance	Sun et al., 2022
DRE	*Populus alba* var. *pyramidalis*	PalERF2	*PalRD20, PalSAG113*	Positive	Positive	6b	Participate in drought tolerance	Chen N. N. et al., 2022
DRE/CRT	*Prunus persica*	PpERF61	*PpTPS1, PpTPS3*	Positive	Positive	3a	Regulate linalool biosynthesis	Wei et al., 2022
ATCTA	*Arabidopsis thaliana*	RAP2.12	*luc*	Positive	Positive	8b	Participate in hypoxia-responsive	Zheng H. et al., 2021
DRE/CRT	*Solanum lycopersicum*	SlDREBA4	*Hsp*	Positive	Positive	3e	Participate in heat tolerance	Mao et al., 2020
DRE/CRT	*Solanum lycopersicum*	Sl-ERF.B3	*Sl-IAA27*	Positive	Positive	6b	Integrates ethylene and auxin signaling	Liu et al., 2018
GCC box	*Salvia miltiorrhiza*	SmERF73	*DXR1, CPS1, KSL1, CYP76AH3*	Positive	Positive	8b	Regulate tanshinone biosynthesis	Zheng H. et al., 2021
GCC box	*Triticum aestivum*	TaERF3	*BG3, Chit1, RAB18, LEA3, TIP2, POX2, GST6*	Positive	Positive	6b	Participate in salt and drought tolerance	Rong et al., 2014
DRE/CRT,GCC box	*Nicotiana tabacum*	Tsi1	unknow	/	Positive	7a	Regulate pathogen infection, Participate in salt tolerance	Park et al., 2001
DRE/CRT,GCC box	*Solanum lycopersicum*	TSRF1	*PR1, PR2, PR3/*unkown	Positive/Negative	Positive	6e	Regulate pathogen infection, Participate in osmotic and drought tolerance	Zhang et al., 2004; Quan et al., 2010
GCC box	*Vitis. amurensis *'Shuang You'	VaERF16	*VaPDF1.2*	Positive	Positive	8b	Participate in resistance to Botrytis cinerea infection	Zhu et al., 2022
DRE/CRT	*Zoysia japonica*	ZjDREB1.4	unknow	/	Positive	3d	Participate in temperature tolerance	Feng et al., 2019
DRE/CRT	*Zea mays*	ZmDREB1A	*KIN1, KIN2, COR15A*	Positive	Positive	3d	Participate in dehydration and cold tolerance	Qin et al., 2004
GCC box	*Zea mays*	ZmERF061	unknow	Positive	Positive	6b	Participate in resistance to Exserohilum turcicum infection	Zang et al., 2021
GCC box-like motif	*Arabidopsis thaliana*	RRTF1/ERF109	*ZAT12*	Positive	Positive	8c	Participate in ROS tolerance	Wang et al., 2020

## Regulatory mechanism of ERFs involved in their transcriptional activations

ERFs recognize specific motifs and function as activator or repressor of a particular gene. In general, activation domains identified in plant. ERFs do not have distinct sequence motifs but tend to be rich in acidic amino acids, like glutamic acid, aspartic acid. Tiwari et al. (2012) named a motif ‘EDLL’ based on the conserved glutamic acid (E), aspartic acid (D) and leucine (L) residues. The EDLL motif has the ability to activate the transcription process. For example, ORA59, an ERF from Arabidopsis, contains EDLL motif and the specific Leu residue at position 228 of the ORA59 EDLL motif mainly contributed to its transcriptional activity on *AtACT* gene expression (Pre et al., 2008). On the other hand, ERFs containing the ERF-associated amphiphilic repression (EAR) motif (LxLxLx or DLNxxP) are usually involved in repression mechanism (Ohta et al., 2001; Hiratsu et al., 2003). For instance, Liu J. X. et al. (2021) demonstrated that AgERF8, an EAR-type ERF from celery (*Apium graveolens*), negatively affected the resistance of transgenic Arabidopsis to ABA and salt stress through inhibiting downstream expression of genes.

**Figure 3:**
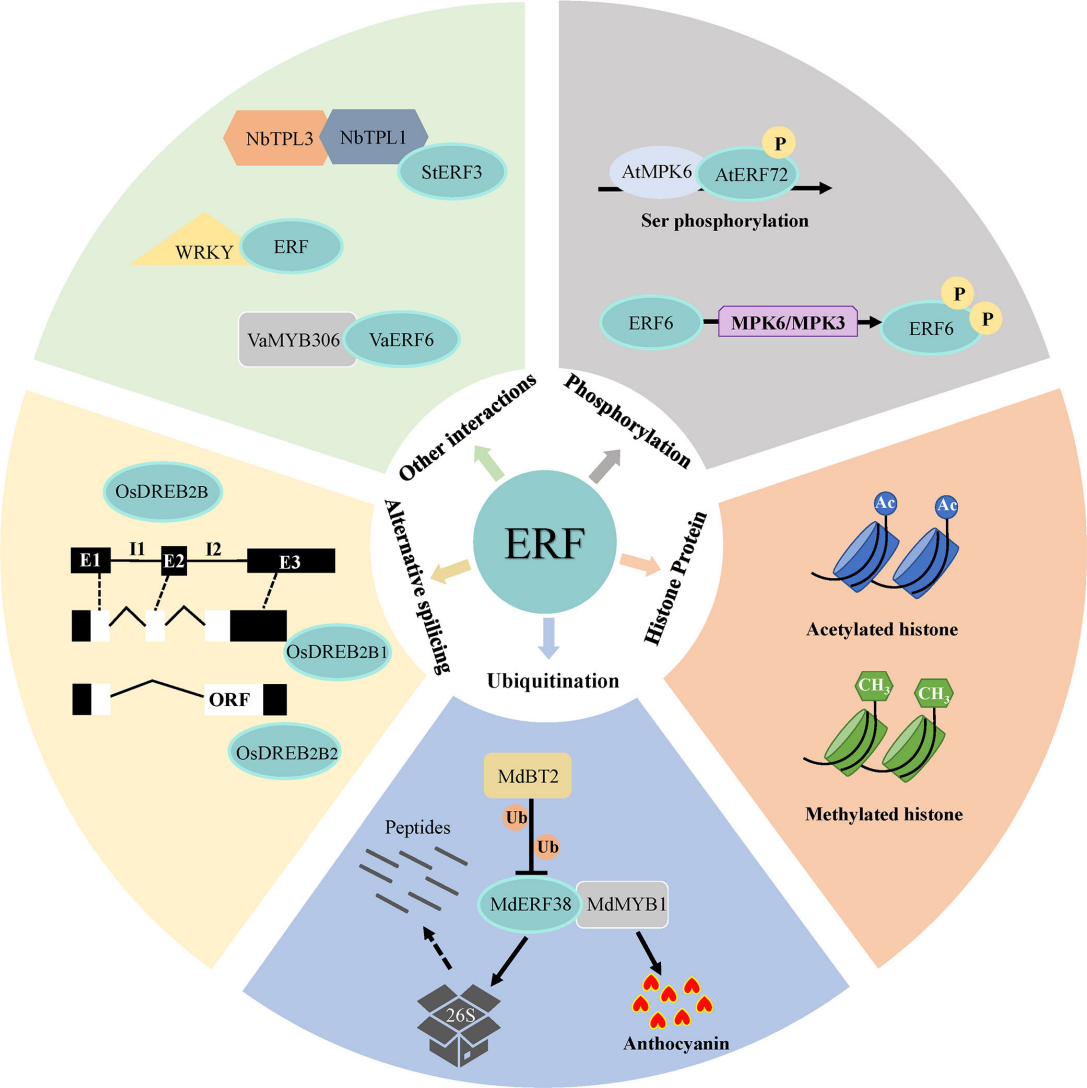
Different regulatory patterns of ERF transcription activation. Regulatory mechanisms of ERFs involved in their transcriptional activations include alternative splicing of their coding mRNA, activation or degradation by post-translational modifications (phosphorylation or ubiquitination), induction by chromatin configurations (such as acetylated- or methylated-histone), and regulation through combination with other nucleic proteins.

The transcriptional activity of ERFs might also be affected by post-translational modified histone, such as acetylated- or methylated-histone (**Figure 3**). These modifications activate or repress transcription by generating more ‘open’ or ‘closed’ chromatin configurations, respectively (Pfluger and Wagner, 2007). A study in peanut (*Achnids hypogaea*) showed that inhibition of histone deacetylase (HDACs) and polyethylene glycol (PEG) treatment induced acetylation around the promoter region of *AhDREB1*, which promoted the transcription of *AhDREB1* and improved the drought resistance in plant (Zhang B. H. et al., 2018). Anh Tuan et al. (2016) suggested that methylated histone modification induced the expression of *PpEBB* and regulated bud break in Japanese pear (*Pyrus pyrifolia*) by activating cell cycle regulatory genes.

Post-translational modifications (PTMs) influence ERF transcriptional activity through a series of ways including phosphorylation and ubiquitination (**Figure 3**). Protein phosphorylation is one of the reversible PTM of ERFs, which are controlled by kinases to phosphorylate and phosphatases to dephosphorylate substrates. MAPKs or MPKs (Mitogen-activated protein kinases), a kind of kinases, are involved in phosphorylating protein substrates to regulate cellular processes (Lee et al., 2015). For instance, Park H. C. et al. (2021) suggested that the phosphorylation of AtERF72 by AtMPKs plays roles at increased DNA binding activity and many stress signaling pathways, including heat and oxidative stress in Arabidopsis. Similarly, phosphorylation of Arabidopsis ERF6 by MPK3/MPK6 in either the gain-of-function transgenic plants or in response to *Botrytis cinereal* infection increases ERF6 protein stability (Meng et al., 2013). Ubiquitination is another essential PTM that affects the structure or stability of substrate proteins. In rice, the E3-ubiquitin ligase OsHOS1 targets OsEREBP1 and OsEREBP2 for degradation and modulates the expression of *OsRMC*, a gene involved in root mechanosensing, through the interaction with two ERFs (Lourenco et al., 2015). Besides, An et al. (2021) revealed that MdBT2 negatively modulated MdERF38-promoted anthocyanin biosynthesis by accelerating the ubiquitination-mediated degradation of the apple (*Malus pumila*) MdERF38 protein in response to drought stress.

Moreover, alternative splicing has been reported to impact the transcriptional activity of ERFs (**Figure 3**). Various ERF functional isoforms produced by alternative splicing were found, such as, rice OsDREB2A/2B (Matsukura et al., 2010), maize (*Zea mays*) ZmDREB2A (Qin et al., 2007), wheat (*Triticum aestivum*) WDREB2 (Egawa et al., 2006), and barley (*Hordeum vulgare*) HvDRF1 (Xue and Loveridge, 2004). It was recently revealed that plants could produce an inactive ERF form containing stop codons before the DNA binding domain during normal conditions, while under stress conditions, the exon with a premature stop codons is excluded to generate a functional transcription factor (Xie et al., 2019b).

ERFs are also capable of forming transcriptional complex (**Figure 3**). Interaction between these TFs are integral to transcriptional regulation. Several ERFs cooperatively recruit transcriptional co-repressors such as topless (TPL) and topless-related (TPR) (Causier et al., 2012), thereby inhibiting the expression of downstream target genes. In transgenic *Nicotiana benthamiana*, StERF3 from potato (*Solanum tuberosum*) interacted with the co-repressors NbTPL1 and NbTPL3 *via* the EAR motif, which facilitated the cell death (Qi et al., 2022). On the other hand, it was found that protein-protein interactions between ERF and WRKY and synergistic regulatory effects in Arabidopsis and Persimmons (*Diospyros kaki* Thunb) (Zhu et al., 2019). In addition, Zhu et al. (2022) demonstrated that VaERF16 from *Vitis amurensis* ‘Shuang You’ interacted with the MYB family transcription factor VaMYB306, and the VaERF16-VaMYB306 transcriptional complex resulted in higher transcript levels of *VaPDF1.2* to enhance resistance of grapevine to *Botrytis cinerea* infection.

## Transcriptional regulation of ERFs in response to abiotic stresses

**Figure 4:**
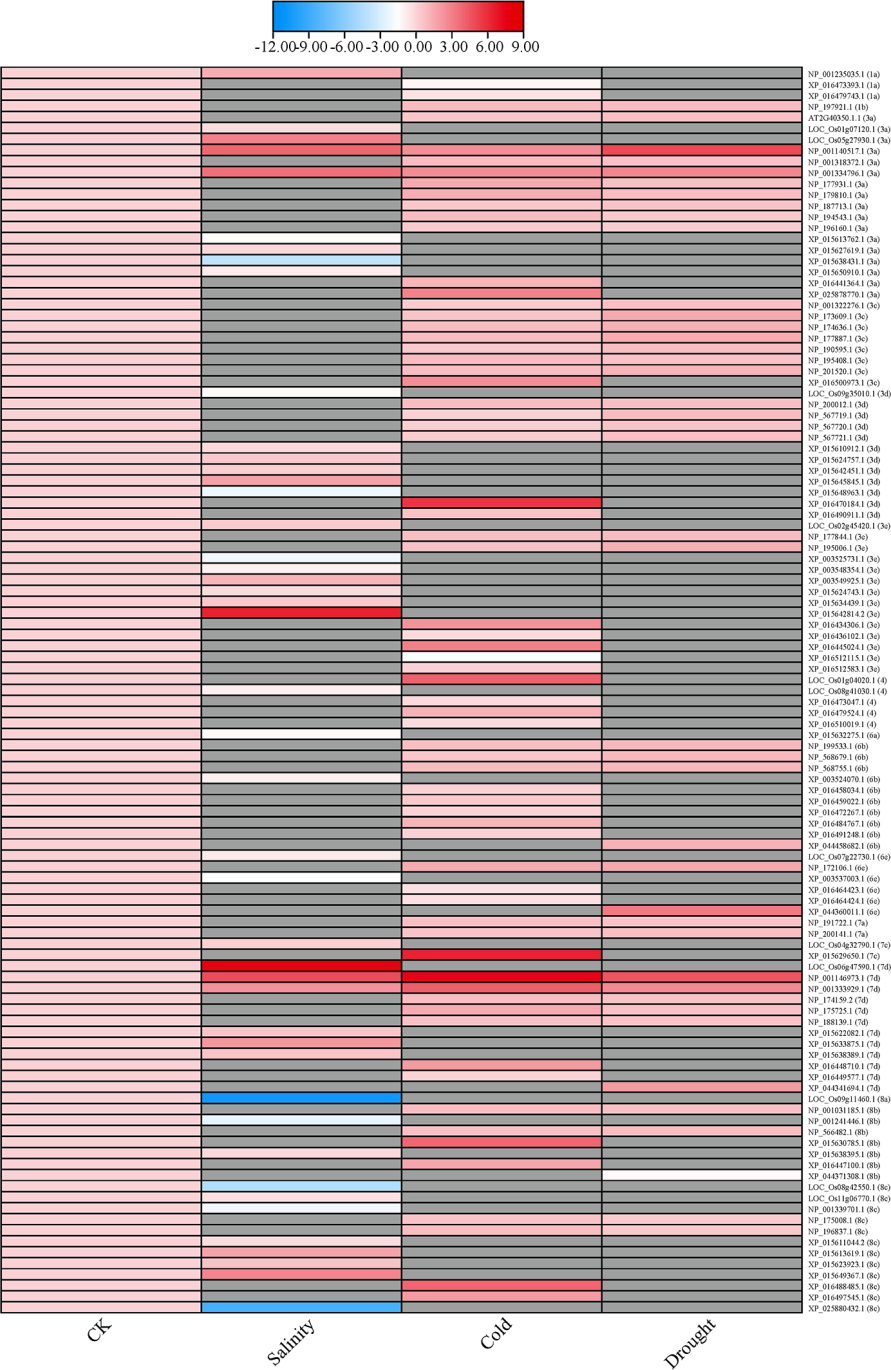
Heatmap of differentially expressed *ERF* genes in response to abiotic stresses. The heatmap was constructed by TBtools (Chen C. J. et al., 2020) with the 113 *ERF* genes transcriptome values of log2FoldChange (Log2FC) in six species (*Arabidopsis thaliana* L., *Glycine max* L., *Nicotiana tabacum* L., *Oryza sativa* L., *Triticum aestivum* L., and *Zea mays* L.) under drought, cold, and salinity stress, respectively (Shankar et al., 2016; Jin et al., 2017; Li et al., 2017; Sharma et al., 2018; Guan et al., 2019; Sharmin et al., 2020; Liu X. et al., 2021; Yu et al., 2022) (**Supplemental Table S5**).

Previous study demonstrated by high-throughput that plenty of stresses, including drought, salinity, cold have a significant induction/repression effect on the transcriptional expression of *ERF* genes, which proved *ERF* subfamily genes are extensively involved in a variety of adversity responses to stress. We collected the transcriptomic data in six plant species to verify our hypothesis on the relevance that *ERF* members of specific subgroup involved in particular abiotic stresses, such as salinity, cold and drought (**Figure 4**). The heatmap was generated by the software TBtools (Chen C. J. et al., 2020) with the 113 *ERF* genes transcriptome values of log2FoldChange (Log2FC) in six species under drought, cold, and salinity stress, respectively (Shankar et al., 2016; Jin et al., 2017; Li et al., 2017; Sharma et al., 2018; Guan et al., 2019; Sharmin et al., 2020; Liu X. et al., 2021; Yu et al., 2022) (**Supplemental Table S5**). Expression profiles of expressed genes are presented with gradient blue and red boxes, blue represents low expression and red represents high expression. Specially, grey boxes indicate no available values (NA) been found.

Results showed that 113 *ERF* genes, from subgroups 1, 3, 4, 6, 7 and 8, were found to be induced or inhibited by these three abiotic stresses. Under salt stress, 45 *ERF* genes in subgroups 1a, 3a, 3d, 3e, 4, 6a, 6b, 6e, 7c, 7d, 8a, 8b and 8c exhibited with differentially expression compared to those under control condition (CK). Among them, the transcriptional level of members in subgroups 1a and 7c were elevated, while those of members in subgroups 4, 6a, 6b and 8a were declined. Under cold stress, only 8 out of 68 *ERF* genes expression reduced, which were distributed in 1a, 3e, 4, 6e and 7d subgroups. There were 3 *ERF* genes from subgroups 3e and 6b without differently expressed. Under drought stress, 42 *ERF* genes distributed in 1b, 3a, 3c, 3d, 3e, 6b, 6e, 7a, 7d, 8b and 8c subgroups, only a single *ERF* of those gene from subgroup 8b decreased in expression. On the other hand, *ERF* in subgroups 3c, 6b, 6d and 7 participate in multiple stresses, while *ERF* in subgroups 6a and 8a focus on only one stress. However, *ERFs* in subgroups 2b, 3b, 5, 6c and 7b have not been found to regulate these abiotic stresses response.

Interestingly, there are only 7 *ERFs*, out of the abovementioned 113 *ERF* genes, have been annotated with biological function involved in abiotic stresses. For example, CBF1 was found with increased transcription level, consistent with its positive role in regulating plant tolerance to cold stress (Lee et al., 2021). Similarly, *CBF2*, *CBF3* and *ERF74* also established mutually confirmed relationships between molecular mechanisms and transcriptome. Meanwhile, it also indicated that there are a large number of *ERFs* (106 out of abovementioned 113) related to abiotic stress, but no specific molecular mechanism researches of them have been carried out.

## ERFs regulate plant responses to various abiotic stresses

**Figure 5:**
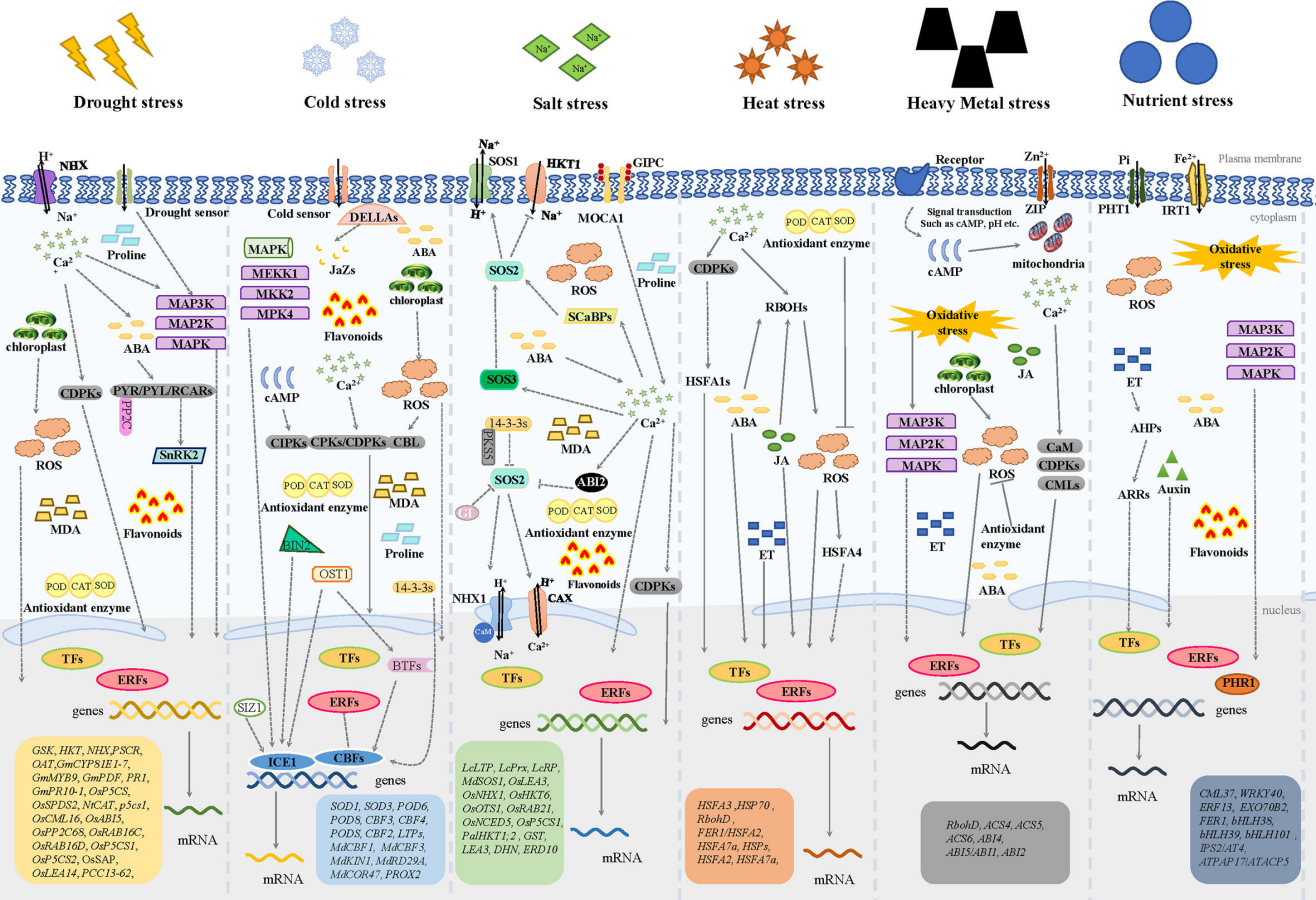
The mechanisms of ERF subfamily transcription factors on regulating plant responses to abiotic stresses. NHX: Na^+^/H^+^ antiporter; MAP3K: Mitogen-activated protein kinase kinase kinases; MAP2K: Mitogen-activated protein kinase kinases; MAPK: mitogen-activated protein kinase; ABA: abscisic acid; CDPK: Ca^2+^-dependent protein kinase; PYR/PYL/RCARs: pyrabactin resistance/pyr1-like/regulatory components of ABA receptors; SnRK2: sucrose non-fermenting-1-related protein kinase 2; PP2C: Type 2C protein phosphatase; ROS: reactive oxygen species; MDA: Malondialdehyde; POD: Peroxidase; CAT: catalase; SOD: Superoxide Dismutase; DELLAs: D-aspartic acid, E-glutamic acid, L-leucine, A-alanin; JaZs: Jasmonate ZIM-domain; MEKK1: Mitogen-activated protein kinase kinase kinases 1; MKK2: Mitogen-activated protein kinase kinases 2; MPK4: mitogen-activated protein kinase 4; cAMP: Cyclic adenosine monophosphate; CIPKs: CBL-interacting protein kinases; CPKs: Ca^2+^-dependent protein kinases; CBL: calcineurin B-like protein; BIN2: brassinosteroid-insensitive 2; OST1: stomatal opening factor 1; 14-3-3s: general regulatory factor, GRF; BTFs: basic transcription factors; SIZ1: SUMO E3 ligase 1; ICE1: Inducer of CBF Expression 1; CBFs: C-repeat binding factors; SOS1, SOS2, SOS3: salt overly sensitive 1, 2, 3; HKT1: high-affinity potassium transporter 1; MOCA1: monocation induced Ca^2+^ increases 1; GIPC: glycosyl inositol phosphorylceramide; SCaBPs: SOS3-like calcium binding proteins; ABI2: ABA insensitive 2; PKS5: SOS2-like Protein Kinase 5; GI: Gigantea; CAX: Cation/H^+^ exchanger antiporter; CaM: calmodulin; CMLs: calmodulin-like proteins; RBOHs: respiratory burst oxidase homologs; HSF: HEAT SHOCK FACTOR (e.g., HSFA1s, HSFA4); JA: Jasmonic acid; ET: Ethylene; PHT1: Phosphate Transporter 1; IRT1: Iron-regulated transporter 1; ZIP: ZRT/IRT-Related Protein; AHPs: Arabidopsis Histidine-containing Phosphotransmitters; ARRs: Arabidopsis Response Regulators; PHR1: phosphate starvation response 1.

To withstand environmental stresses, plants have evolved interconnected regulatory pathways that enable them to respond and adapt to their environments in a timely manner (Zhang H. et al., 2022) (**Figure 5**) (Agafonov et al., 2016; Nath and Tuteja, 2016; Yuan et al., 2016; Lievens et al., 2017; Zargara et al., 2017; Abhinandan et al., 2018; Dutta et al., 2018; Tiwari and Lata, 2018; Priya et al., 2019; Gong et al., 2020; Mahmood et al., 2020; Angulo et al., 2021; Devireddy et al., 2021; Jha et al., 2022). Plant response strategies to abiotic stresses involve changes at the molecular, cellular, biochemical, and physiological levels (Baillo et al., 2019). The response strategies could be classified to non-adaptive responses and adaptive responses, former including the detrimental changes in membrane fluidity and protein structure as well as the disruptions in enzyme kinetics and molecular interactions, latter including the repair of stress-induced damage, the re-balancing of cellular homeostasis and the adjustment of growth to levels suitable for the particular stress condition (Zhu, 2016; Zhang et al., 2020).

Increasing studies support the involvement of ERFs in abiotic stress, including drought, salinity, cold, high temperature, heavy metal toxicity and nutrition stresses which responses by controlling the activation of stress-response genes (Klay et al., 2018; Debbarma et al., 2019). To uncover the potential roles of ERFs from different subgroups in different abiotic stresses, most of the functionally annotated ERF TFs were categorized into the above-mentioned 8 subfamilies (**Figure 1** and **Table 2**). More than half of the ERFs subfamilies are involved in abiotic stress responses. Among them, the six subfamilies 2, 3, 4, 6, 7 and 8 are involved in drought, salt, cold and other stress responses, and their detailed subgroups are 2a, 3a, 3c, 3d, 3e, 4, 6b, 6d, 6e, 7a, 7d, 8b and 8c. Most of the drought responsive members were from 6b and 8b. The great majority of ERFs related salinity were distributed in 6b subgroup. The ERFs subgroup mainly involved in clod stress response were 3d and 6b. In addition, five subgroups were found to regulate the heat tolerance of plants, including 3e, 4, 6d, 6e and 8b. ERFs of subgroups 6d and 8b are involved in the response to heavy metal toxicity. There are three subgroups of ERFs participating in nutrition stress, including subgroups 3a, 6d, and 8c. In short, ERFs of subgroups 3, 6 and 8 seem to participate in most kinds of abiotic stresses, such as drought, salinity, cold, heat and nutrition stresses.

**Table 2:** ERF transcription factors involved in plant abiotic stress response.

Species	Stress response	Nomenclature	Subgroup	Target gene	Regulation of target gene	Regulation of biological response	Function	References
*Brassica oleracea* var*. italica Plenck*	Salt	BoERF1	6e	unknow	\	Positive	Participate in salt stress response	Jiang et al., 2019
*Betula platyphylla*	Cold	BpERF13	6b	*SOD1, SOD3, POD6, POD8, CBF3, CBF4*	Positive	Positive	Participate in cold stress response	Lv et al., 2019
*Cynodon dactylon*	Cold	CdERF1	2a	*PODS, CBF2, LTPs*	Positive	Positive	Participate in cold stress response	Hu et al., 2020
*Heuchera villosa*	Drought/salt	ERF1-V	8b	*GSK, HKT, NHX, PSCR, OAT*	Positive	Positive	Participate in drought and salt stress response	Xing et al., 2017
*Glycine max*	Drought	GmDREB1	3c	*GmCYP81E1-7, GmMYB9, GmPDF*	Positive	Positive	Participate in drought stress response	Chen N. et al., 2020
*Glycine max*	Drought	GmERF113	8c	*PR1, GmPR10-1*	Positive	Positive	Participate in drought stress response	Fang et al., 2022
*Lycopersicon esculentum*	Drought	JERF1	8b	*OsP5CS, OsSPDS2*	Positive	Positive	Participate in drought stress response	Zhang et al., 2010
*Solanum lycopersicum*	Cold/drought	JERF3	8b	unknow	Positive	Positive	Participate in cold and drought stress response	Wu et al., 2008
*Lotus corniculatus*	Salt	LcERF056	6b	*LcLTP, LcPrx, LcRP*	Positive	Positive	Participate in salt stress response	Wang D. et al., 2021
*Malus baccata*	Cold	MbERF11	3c	unknow	Positive	Positive	Participate in cold stress response	Han et al., 2020
*Malus × domestica*	Cold	MdABI4	3a	*MdCBF1 , MdCBF3, MdKIN1, MdRD29A, MdCOR47*	Positive	Positive	Participate in cold stress response	An et al., 2022
*Malus × domestica*	salt	MdERF106	6b	*MdSOS1*	Positive	Positive	Participate in salt stress response	Yu et al., 2020
*Malus × domestica*	Drought	MdERF38	3c	unknow	Positive	Positive	Participate in drought stress response	An et al., 2020
*Medicago falcata*	Cold	MfERF1	8b	*PROX2*	Negative	Positive	Participate in cold stress response	Zhuo et al., 2018
*Nicotiana tabacum*	Drought	NtERF172	3c	*NtCAT*	Positive	Positive	Participate in drought stress response	Zhao et al., 2020
*Oryza sativa*	Cold	OsBIERF3	6b	unknow	\	Negative	Participate in cold stress response	Hong et al., 2022
*Oryza sativa*	Cold	OsERF096	6b	unknow	Positive	Positive	Participate in cold stress response	Sun et al., 2022
*Oryza sativa*	Drought/heat	OsERF115/AP2EREBP110	4	*p5cs1*	Positive	Positive	Participate in heat and drought stress response	Park H. C. et al. (2021)
*Oryza sativa*	Salt	OsERF19	3c	*OsLEA3, OsNHX1, OsHKT6, OsOTS1, OsRAB21, OsNCED5, OsP5CS1*	Positive	Positive	Participate in salt stress response	Huang S. et al., 2021
*Oryza sativa*	Drought	OsERF71	8b	*OsABI5, OsPP2C68, OsRAB16C, OsRAB16D, OsP5CS1, OsP5CS2*	Positive	Positive	Participate in drought stress response	Li J. J. et al., 2018
*Oryza sativa*	Drought	OsERF83	6e	*OsSAP, OsLEA14, PCC13-62*	Positive	Positive	Participate in drought stress response	Jung et al., 2021
*Oryza sativa*	Salt	OsERF922	6d	unknow	Positive	Negative	Participate in salt stress response	Liu et al., 2012
*Populus alba* var. *pyramidalis*	Salt	PalERF109	8c	*PalHKT1;2*	Positive	Positive	Participate in salt stress response	Chen N. et al., 2020
*Populus alba* var. *pyramidalis*	Drought	PalERF2	6b	*PalRD20, PalSAG113*	Positive	Positive	Participate in drought stress response	Chen N. et al., 2020
*Poncirus trifoliata*	Cold	PtrERF108	8c	*PtrRafS*	Positive	Positive	Participate in cold stress response	Khan et al., 2021
*Poncirus trifoliata*	Cold	PtrERF9	7d	*PtrGSTU17, PtrACS1*	Positive	Positive	Participate in cold stress response	Zhang Y. et al., 2022
*Solanum lycopersicum*	Drought/salt	SlERF84	8c	*AtRD22, AtRD29A, atp5cs1*	\	Positive	Participate in salt and drought stress response	Li J. J. et al., 2018
*Triticum aestivum*	Drought/salt	TaERF3	6b	*BG3, LEA3, DHN, RAB18, SDR, TIP2, Chit1, POX2, OxOx2, GST6*	Positive	Positive	Participate in salt and drought stress response	Rong et al., 2014
*Vitis amurensis*	Cold	VaERF080	6b	*CBF1, CBF2, ICE1, ZAT12, KIN1, SIZ1, RD29A, COR15A, COR47*	Positive	Positive	Participate in cold stress response	Sun et al., 2018
*Vitis amurensis*	Cold	VaERF087	6b	*CBF1, CBF2, ICE1, ZAT12, KIN1, SIZ1, RD29A, COR15A, COR47*	Positive	Positive	Participate in cold stress response	Sun et al., 2018
*Vitis amurensis*	Cold	VaERF092	6b	*VaWRKY33*	Positive	Positive	Participate in cold stress response	Sun et al., 2019
*Vigna angularis*	Salt	VaERF3	7d	*GST, LEA3, DHN, ERD10*	Positive	Positive	Participate in salt stress response	Li et al., 2020
*Arabidopsis thaliana*	Heat/salt/drought	AtERF1	6e	HSFA3, HSP70	Positive	Positive	Participate in heat, salt and drought stress response	Cheng et al., 2013
*Arabidopsis thaliana*	Heat/Aluminum/drought	AtERF74	8b	RbohD	Positive	Positive	Participate in heat, aluminum and drought stress response	Yao Y. et al., 2017
*Arabidopsis thaliana*	Heat/iron	AtERF95	6d	FER1/HSFA2, HSFA7a, HSPs	Positive	Positive	Participate in heat stress and Fe-deficiency response	Sun et al., 2020; Huang S. et al., 2021
*Arabidopsis thaliana*	Heat	AtERF97	6d	HSFA2, HSFA7a, HSPs	Positive	Positive	Participate in heat stress response	Huang S. et al., 2021
*Arabidopsis thaliana*	iron	AtERF109	8c	CML37, WRKY40, ERF13, and EXO70B2	Positive	\	Participate in Fe-deficiency response	Yang et al., 2022
				FER1, bHLH38, bHLH39, bHLH101	Negative	\	Participate in Fe-deficiency response	
*Glycine soja*	Aluminum	GsERF1	6d	ACS4, ACS5, ACS6, ABI4, ABI5/ABI1, ABI2	Positive/Negative	Positive	Participate in aluminum stress response	Li T. et al., 2022
*Jatropha curcas*	Phosphorus	JcERF035	3a	IPS2/AT4 , ATPAP17/ATACP5	Negative	Negative	Participate in P-deficiency response	Chen et al., 2018

### ERFs involved in drought stress

Drought is one of the most serious abiotic stresses that could adversely hinder plant growth, development and productivity (Ali et al., 2021). Drought stress usually causes water deficit in plant, which is embodied in height decreased, leaf wilting, number and area of leaves changed (Yang X. Y. et al., 2021). At the physiological and biochemical level, the balance of ROS homeostasis in plant was broken down due to the excessive accumulation (Zhao J. Q. et al., 2021). Furthermore, the increasing reactive oxygen free radicals make plant cells suffer oxidative stress (Guo et al., 2018; Yang X. Y. et al., 2021).

It seems that ERFs could modulate the transcriptional expression of drought-responsive gene for production of osmolyte or regulate the plant ability on ROS scavenging (**Table 2**). On the one hand, ERFs modulate the expression of osmolyte synthesis genes to confer drought tolerance (Wang et al., 2018). Zhang et al. (2010) found that JERF1, a tomato ERF protein, significantly enhanced drought tolerance of transgenic rice through increasing the synthesis of the osmolyte proline. Besides, An et al. (2020) found that MdERF38, an apple ERF protein, promoted anthocyanin biosynthesis in response to drought stress. In rice, *OsERF115/AP2EREBP110* enhance drought tolerance by elevating the expression level of a proline biosynthesis *P5CS1* gene (Park S. I. et al., 2021). In wheat, Rong et al. (2014) showed that TaERF3 positively regulated stress-related genes which increasing the accumulation of proline and chlorophyll thereby enhancing the drought tolerance. ERF1-V in wheat (*Heuchera villosa*) improved drought tolerance *via* modulating *P5CR* and *OAT* involved in the proline synthesis (Xing et al., 2017). GmDREB1 could confer drought tolerance of soybean by increasing the photosynthetic efficiency, the accumulation of osmoregulation substances, and the synthesis of melatonin (Chen K. et al., 2022). On the other hand, ERFs could also enhance the ROS scavenging ability of plants under drought condition. Drought induced *NtERF172* was proven to positively promote the catalase (CAT)-mediated hydrogen peroxide scavenging in tobacco (Zhao et al., 2020). *SlERF84*-overexpressed tomato could elevate both superoxide dismutase (SOD) and peroxidase (POD) activities under drought stress (Li Z. et al., 2018).

Recent studies showed that ERFs regulate the plant drought-tolerant responses mainly through the abscisic acid (ABA) signaling pathways (**Table 2**). ABA is responsible for drought stress tolerance *via* its capacity to enhance stomatal closure and regulate the expression of drought stress-responsive genes (Takahashi et al., 2020). For instance, the expression of the ERF-type transcription factor OsERF83 was induced by ABA, and that rice overexpressing OsERF83 showed a stronger drought tolerance (Jung et al., 2021). PalERF2 from poplar is recruited to up-regulate the transcription of *PalRD20* (a stress-inducible caleosin and positively regulates stomatal closure) and down-regulate the expression of *PalSAG113*, a repressor of the ABA pathway, resulting in enhanced tolerance to drought (Chen N. N. et al., 2022). Li J. J. et al. (2018) demonstrated that OsERF71 played a positively affect drought tolerance of rice by enhancing the expression of genes (such as *OsABI5*, *OsPP2C68*, *OsRAB16C* and *OsRAB16D*) associated with ABA signaling and proline biosynthesis. Fang et al. (2022) recently showed that the GmERF113 improves the drought tolerance of soybean by downregulating the *abscisic acid 8’-hydroxylase 3* (*GmABA8’-OH 3*), associated with upregulating of SOD and POD activities.

These researches mentioned above indicate that a large number of ERFs regulate plant responses to drought in ABA-dependent manner. Through the ABA pathway, plants could then synthesize series of osmolyte and accumulate various ROS-scavenging enzymes.

### ERFs involved in salinity stress

Salinity is a widespread abiotic stress that constrains plant growth (Wang D. et al., 2021). Under salt stress, plants suffer osmotic stress and ionic toxicity (Wang H. et al., 2022). Osmotic and ionic stresses further exert detrimental effects on plants, such as oxidative stress (Yang and Guo, 2018). Recently, many ERFs have been found to improve plant tolerance to salinity (**Table 2**).

Many ERFs could regulate plants’ tolerance to salinity by modulating Na^+^/K^+^ homeostasis. In rice, OsERF922 might negatively regulated plant tolerance to salt stress by destroying Na^+^/K^+^ homeostasis and mediating ABA-signaling pathway (Liu et al., 2012), while OsERF19 could regulate the expression of the salt-responsive Na^+^/H^+^ antiporter *OsNHX1* and the high-affinity K^+^ transporter *OsHKT6* and *OsOTS1* (Huang S. et al., 2021). Besides, PalERF109 enhanced poplar salt tolerance through upregulating a high-affinity K^+^ transporter (HKT) gene *PalHKT1;2* (Chen N. et al., 2020). In addition, MdERF106 associated with MdMYB63, promoted the expression of downstream *MdSOS1* and further improved the Na^+^ expulsion under salt stress in apple (*Malus × domestica*) (Yu et al., 2020).

ERFs could also regulate the expression of antioxidant enzyme for scavenging salinity induced ROS. The positive regulator LcERF056 is found to enhance salt tolerance in *Lotus corniculatus* by directly upregulating ROS-related genes *LcLTP*, *LcPrx*, and *LcRP* (Wang D. et al., 2021). In broccoli (*Brassica oleracea* var. *italica Plenck*), BoERF1 significantly reduced the content of H_2_O_2_ and increased the activities of CAT, POD and SOD, thereby improving salt resistance of plant (Jiang et al., 2019). In transgenic Arabidopsis, overexpression of *VaERF3* from *Vigna angularis* resulted in higher levels of proline accumulation and lower malondialdehyde (MDA) and ROS contents under salinity stress conditions (Li et al., 2020).

Thus, ERFs are involved in modulating the salt tolerance of plant by modulating Na^+^/K^+^ homeostasis and regulating the expression of antioxidant enzyme. But the stress-related molecular regulatory network is known to complex and mostly unexplored, the various roles of ERFs in maintaining ion homeostasis or metabolic balance in plants under salt stress may need further concern.

### ERFs involved in cold stress

Cold stress, one of the most major abiotic stresses, can be generally categorized into chilling and freezing (Li W. Y. et al., 2022). Both of them usually reduce the fluidity of cell membrane, affect the stability of proteins, break the intracellular ion homeostasis in plant (Ding et al., 2019). Furthermore, the burst of ROS caused by cold produced the osmotic stress and oxidative stress, which result in cell damage and even death (Hu et al., 2020). Actually, plants have evolved sophisticated mechanisms (**Table 2**) to withstand cold stress (Zheng S. et al., 2021).

Increasing studies have demonstrated that ERFs regulate the activities of antioxidant enzyme and ROS-scavenging to change the tolerance of cold in plant. Wu et al. (2008) demonstrated that JERF3 reduced the accumulation of ROS, which enhanced adaptation to freezing in tobacco. Sun et al. (2018) showed that both VaERF080 and VaERF087 increased antioxidant enzyme activities and regulated the expression of cold-related genes *CBF1*, *CBF2*, *ICE1*, *ZAT12*, *KIN1*, *SIZ1*, *RD29A*, *COR15A*, and *COR47*, which improved the cold tolerance in transgenic Arabidopsis. Lv et al. (2019) found that overexpression of *BpERF13* up-regulated *SOD1*, *SOD3*, *POD6*, *POD8*, *CBF3* and *CBF4* genes and down-regulated the accumulation of ROS to resist oxidative stress, thus enhancing the cold tolerance of birch (*Betula platyphylla*). Hu et al. (2020) showed that overexpression of *CdERF1* in bermudagrass (*Cynodon dactylon*) positively regulated cold response by activating cold stress-related genes *PODs*, *CBF2* and *LTPs*. In transgenic Arabidopsis, heterologous expression *Malus baccata* MbERF11 contributed to cold stress response probably by promoting the ability to scavenge ROS (Han et al., 2020). Zhang Y. et al. (2022) revealed that (*Poncirus trifoliata*) *PtrERF9* acted downstream of ethylene signaling and functioned positively in cold tolerance *via* modulation of ROS homeostasis by regulating *PtrGSTU17* gene. Moreover, varieties of physiological and biochemical reactions could be regulated by ERFs to influence the adaptation of cold in plant. Zhuo et al. (2018) suggested that MfERF1 from *Medicago falcata* conferred cold tolerance through polyamine turnover, antioxidant protection and proline accumulation. Khan et al. (2021) revealed that PtrERF108, a positive regulator of cold tolerance, is attributed to its role in the modulation of raffinose content by transcriptionally regulating the *PtrRafS* gene. In rice, OsBIERF3 significantly decreased the contents of proline to suppress the cold stress response (Hong et al., 2022).

Besides, ERFs neutralize the damage of cold stress to plant through hormone signaling pathways. Sun et al. (2019) revealed that VaERF092 regulated the transcriptional expression of *VaWRKY33* and further enhanced cold stress tolerance of grape by regulation of hormone metabolism. Sun et al. (2022) suggested that a new module, the miR1320-OsERF096, regulates cold tolerance of rice by repressing the JA-mediated cold signaling pathway. An et al. (2022) found that MdABI4 integrated jasmonic acid and abscisic acid signals to precisely modulate cold tolerance in apple through the JAZ-ABI4-ICE1-CBF regulatory cascade.

These researches mentioned above attest the principal roles of ERFs in plant suffering cold stress, inducing the expression of genes involved in hormone signaling pathways, elevating the activities of antioxidant enzyme and ROS-scavenging. However, it is worth mentioning that the regulatory roles of plant ERFs in cold tolerance are far from clear as only a very few of them have been explicitly characterized, relative to a large number of genes in this superfamily (Khan et al., 2021).

### ERFs involved in other abiotic stresses

Many other abiotic stresses, such as high temperature, heavy metal toxicity and nutrition deficiency, also cause plant growth inhibition, damage, and in the most severe cases, cell death, resulting in major crop yield losses worldwide (Gechev and Petrov, 2020). In order to adapt to these stresses, plants must sense the changes of the temperature, concentrations of heavy metal and mineral nutrient both externally and internally, and generate physiological and morphological responses *via* a series of metabolic processes including scavenging of ROS and biosynthesis of hormones (e.g., ethylene, jasmonic acid).

Among them, high temperature usually decreases the biosynthesis of auxin and cytokinin, and further impede growth and development of plants. Besides, heat stress could induce phase transition of cell membrane and elevate the accumulation of excess ROS, which leads to oxidative stress (Hasanuzzaman et al., 2013a; Hasanuzzaman et al., 2013b; Li and Howell, 2021). The Arabidopsis AtERF1, an upstream component in both jasmonic acid and ethylene signaling, was showed to activate *HSFA3* and *HSP70* expression and enhanced the thermotolerance (Cheng et al., 2013). Yao Y. et al. (2017) demonstrated that AtERF74 directly binds to the promoter of *RbohD* and activates its expression for ROS elimination under heat stresses in Arabidopsis. Under heat stress, AtERF95 can physically interact with AtERF97 for regulating a common set of target genes, including known heat-responsive genes and directly bind to the promoter of HSFA2 (Sun et al., 2020; Huang J. Y. et al., 2021).

Heavy metals, such as aluminum (Al) and zinc (Zn), at elevated concentrations produce severe toxicity symptoms in plants, directly interacting with sulfhydryl group of functional proteins, which disrupts their structure and function, and thus, renders them inactive (Janicka-Russak et al., 2008; Sharma and Dietz, 2009; DalCorso et al., 2013). ERF-VII transcription factors are usually key regulators of the molecular response to hypoxia (van Dongen and Licausi, 2015). Carbonare et al. (2019) found that poplar (*Populus* spp.) ERF-VII Pop_ERFB2-1 could regulate the expression of hypoxia-responsive genes under high intracellular Zn concentrations. Li L. et al. (2022) suggested that overexpression of GsERF1 may enhance aluminum tolerance of Arabidopsis through an ethylene-mediated pathway and/or ABA signaling pathway. Yao Y. et al. (2017) demonstrated that Arabidopsis AtERF74 enhance plant tolerance to aluminum toxicity dependent on the ERF74-RbohD-ROS signal pathway.

14 essential mineral nutrient elements are required for the optimal growth and development of plants, such as phosphorus (Pi) and iron (Fe), etc (White and Brown, 2010). Plants suffered from Phosphorus (P) deficiency will experience a strong reduction of primary root growth and an arrest of cell division as well as the loss of the quiescent center identity (Sanchez-Calderon et al., 2005; Sanchez-Calderon et al., 2006; Svistoonoff et al., 2007). Chen et al. (2018) indicated that down-regulation of the *JcERF035* gene might contribute to the regulation of root system architecture and both biosynthesis and accumulation of anthocyanins in aerial tissues of Arabidopsis under low Pi conditions. Yang et al. (2022) suggested that Arabidopsis AtERF109 is a negative regulator of the leaf response to Fe deficiency. Sun et al. (2020) found that Arabidopsis AtERF95, formed as complex with EIN3, could specifically binds to promoter GCC-box and transactivates of *FER1* expression, and consequently regulate sensitivity to Fe deficiency during seedling establishment.

To uncover the potential roles of ERFs from different subgroups in response to various abiotic stresses. Most of the functionally annotated ERFs were categorized into the abovementioned 23 subgroups. We found that nearly half of the ERF subgroups have not been reported to be involved in any abiotic stress, such as subgroup 1a, 1b, 2b, 3b, 5, 6a, 6c, 7b, 7c and 8a. The remaining subgroups were revealed to participate in response to at least one kind of abiotic stresses. Interestingly, members in subgroup 2a are thought to exclusively regulate cold stress, while members in subgroup 3a, 3c, 3d, 6b, 8b and 8c were found to be able to take over the regulations of drought and cold responses, respectively. Similarly, members in subgroup 3e are recognized to exclusively regulate heat stress and members in subgroup 7a are thought to exclusively modulate salinity, while members in subgroup 6d, 6e and 8b are involved in heat and salt stresses. Meanwhile, Members of subgroup 3c, 6b, 6e and 8b were found to regulate the drought and salt tolerance of plants and members of subgroup 3c, 6b, 7d and 8b are involved in cold and salinity responses. ERF members of 4 subgroups (3c, 6b, 8b, 8c) regulate the molecular mechanism of plant responded to three kinds of stresses, including drought, salt and cold. In addition, ERFs of subgroup 8b seem to participate in most kinds of abiotic stresses, such as drought, salinity, chilling, Heat and heavy metal stress. Moreover, subgroup 6d of ERFs participated in salt, heat, heavy metal and nutrition stress. So, it’s true that the variation of AP2/ERF domain in ERF subfamily affect their biological functions related to abiotic stresses.

## ERFs regulate plant tolerances to abiotic stress mainly through modulation the syntheses of antioxidative metabolites

Adverse conditions such as drought, salinity, cold and other abiotic stresses usually induce the accumulation of ROS that are detrimental to plant growth and development (Miller et al., 2010; Dreyer and Dietz, 2018). Excessive ROS would lead to increased levels of cell death, thus inhibiting plant growth and reducing crop productivity. There are two main ROS scavenging systems that evolved in plants, including enzymatic and non-enzymatic scavenging system. The enzymatic scavenging system is commonly constituted with SOD, ascorbate peroxidase (APX), and glutathione reductase (GR) (Wang C. L. et al., 2021). Besides, plants depend on non-enzymatic pathways to scavenge several highly toxic ROS, such as ^1^O_2_ and ^•^OH, that cannot be scavenged by enzymatic antioxidant systems (Das and Roychoudhury, 2014; Morales and Munne-Bosch, 2019). Generally, the non-enzymatic scavenging system contains several antioxidative metabolites, including MDA and proline, etc. Recently, increasing studies found that flavonoids, a large group of natural metabolites with variable phenolic structures, play crucial roles to scavenge free radical activity to reduce oxidative stress in plants (Pi et al., 2016; Pi et al., 2018; Pi et al., 2019).

Flavonoids represent a wide array of plant secondary metabolites which present C6-C3-C6 structure (Dias et al., 2021). According to their multifarious structures, flavonoids can be further divided into flavanones, flavones, flavonols, isoflavonoids, anthocyanidins and proanthocyanidins (PAs) (Zhao C. N. et al., 2021). In recent years, more and more attention has been paid to the functions of flavonoids. Ferreyra et al. (2012) indicated that flavonoids are involved in plant growth and development processes such as aroma, coloration and signaling (Zhang J. et al., 2009; Jeon et al., 2022; Mahon et al., 2022), while Dong et al. (2020) emphasized that flavonoids also exhibit specific stress resistance function in response to abiotic stresses, such as drought, salinity, cold, heavy metals and other abiotic stresses (Ding et al., 2019; Ghori et al., 2019; Chourasia et al., 2021; Razi and Muneer, 2021). It is worth noting that flavonoids often generated as scavengers to free radicals which always increasingly accumulate in plant suffering abiotic stresses (Zheng et al., 2022). Excessive free radicals are known to severely cause plant death for its strong oxidant effects (Nauser and Gebicki, 2019). Acting as effective antioxidants, flavonoids contribute to eliminating the oxidative free radicals, which is benefiting from the hydroxyl groups in flavonoids (Speisky et al., 2022). Flavonoids biosynthesis is controlled by diverse enzymes, such as CHS (chalcone synthase), CHI (chalcone isomerase), DFR (dihydroflavonol 4-reductase), F3H (flavanone 3-hydroxylase), F3’H (flavonoid 3’-hydroxylase), F3’5’H (flavonoid 3’,5’-hydroxylase), UFGT (UDP-glucose:flavonoid 3-glucosyltransferase), and ANS (anthocyanin synthase) (Fu et al., 2021). It is reported that numerous transcription factors including MYB, ERF, WRKY and bHLH have been found to affect the synthesis of flavonoids by regulating the expressions of these target genes (Ding et al., 2022). In order to reveal the relationship between ERFs and flavonoid biosynthesis, annotated functional ERFs are present in **Table 3**. It was found that ERFs in subgroups 1b, 3a, 3c, 6b, 6e, 7b, 7d, 8b and 8c play crucial roles in the modulation of flavonoids metabolism.

**Table 3:** Regulation of ERFs transcription factors on metabolism of flavonoids.

Species	Nomenclature	Subgroup	Regulation of target gene	Regulation of biological response	Target gene	Function	References
*Citrus reticulata*	CitERF32	3a	Positive	Positive	*CitCHIL1*	Improved activation efficiency and flavonoid accumulation	Zhao C. N. et al., 2021
*Citrus reticulata*	CitERF33	3a	Positive	Positive	*CitCHIL1*	Improved activation efficiency and flavonoid accumulation	Zhao C. N. et al., 2021
*Lilium brownii* var. *Viridulum*	ERF061	3a	Negative	Negative	*LhMYBSPLATTER*	Negative regulation of anthocyanin biosynthesis	Cao et al., 2021
*Lilium brownii* var. *Viridulum*	ERF071-like	8b	Negative	Negative	*LhMYBSPLATTER*	Negative regulation of anthocyanin biosynthesis	Cao et al., 2021
*Lilium brownii* var. *Viridulum*	ERF4	7b	Negative	Negative	*LhMYBSPLATTER*	Negative regulation of anthocyanin biosynthesis	Cao et al., 2021
*Lilium brownii* var. *Viridulum*	ERFWIN1-like	1b	Negative	Negative	*LhMYBSPLATTER*	Negative regulation of anthocyanin biosynthesis	Cao et al., 2021
*Fagopyrum tataricum*	FtERF-EAR3	7d	Negative	Negative	*FtF3H*	Negative regulation of anthocyanin biosynthesis	Ding et al., 2022
*Malus domestica*	MdERF109	8c	Positive	Positive	*MdCHS, MdUFGT, MdbHLH3*	Induces the expression of anthocyanin-related genes and the accumulation of anthocyanins	Ma et al., 2021
*Malus pumila*	MdERF1B	6e	Positive	Positive	*MdMYB11*	Promoted the biosynthesis of anthocyanins and proanthocyanidins	Zhang J. et al. (2018)
*Malus pumila*	MdERF38	3c	Positive	Positive	*MdDFR, MdUF3GT, MdCHI, MdCHS*	Regulation of drought-induced anthocyanin biosynthesis	An et al., 2020
*Pyrus bretschneideri*	PbERF22	3c	Positive	Positive	*PbMYB10, PbMYB10b*	Facilitated the expression of anthocyanin biosynthetic structural and regulatory genes	Wu et al., 2020
*Pyrus* spp.	Pp12ERF96	6b	Positive	Positive	*PpMYB114*	Enhance anthocyanin accumulation	Ni et al., 2019
*Pyrus* spp.	Pp4ERF24	6b	Positive	Positive	*PpMYB114*	Enhance anthocyanin accumulation	Ni et al., 2019
*Pyrus* spp.	PpERF105	6b	Positive	Negative	*PpMYB140*	Inhibited anthocyanin biosynthesis	Ni et al., 2021
*Pyrus bretschneideri*	PyERF3	8c	Positive	Positive	*PyMYB114*	Enhance anthocyanin accumulation	Yao G. F. et al., 2017

ERFs are involved in modulation of flavonoids biosynthesis through the co-regulation of transcription factors, especially by the interaction with MBW (MYB-bHLH-WDR) complex (Kirschner, 2022). The Chinese pear (*Pyrus bretschneideri*) PyERF3 was found to interact with PyMYB114 and its partner PybHLH3 to form a new complex (ERF3-MYB114-bHLH3), hence, to co-regulate anthocyanin biosynthesis (Yao G. F. et al., 2017). Besides, Ni et al. (2019) demonstrated that Pp4ERF24 and Pp12ERF96 promoted anthocyanin biosynthesis in ‘Red Zaosu’ pear (*Pyrus* spp.) *via* enhancing the interaction between PpbHLH3 and PpMYB114 as well as the expression of PpMYB114-induced *PpUFGT* gene. Additionally, the ‘Zaosu’ pear *PbERF22* might regulate anthocyanin biosynthesis by enhancing the activation effects of *PbMYB10* and *PbMYB10b* on the *PbUFGT* promoter (Wu et al., 2020). In apple, Zhang J. et al. (2018) revealed that not only did MdERF1B interact with MdMYB9/11 proteins, but also bound to their promoters to activate the expression of *MdLAR*, *MdANR*, and *MdANS*, which induced PAs and anthocyanin production. In *Citrus reticulata*, CitERF33 formed a transcription complex with CitRAV1 to strongly enhance the flavonoid accumulation efficiency (Zhao C. N. et al., 2021). Ni et al. (2021) indicated that PpERF105 inhibited anthocyanin biosynthesis in pear through activating *PpMYB140* capable of interacting with bHLH3 and bHLH33 to form the repressive PpMYB140/bHLH3 or bHLH33/WD-repeat [M(140)BW] complex. Furthermore, recent studies have revealed that some ERFs alone could bind directly to the promoter of flavonoids biosynthesis genes. Ding et al. (2022) illustrated that FtERF-EAR3 inhibited the expression of *FtF3H* through binding to the GCC-box in *FtF3H* promoter, which decreased flavonoids accumulation in *Fagopyrum tataricum*. Zhao C. N. et al. (2021) demonstrated that CitERF32 and CitERF33 activated the transcription of *CitCHIL1* in *Citrus* and *Arabidopsis* for significantly enhancing the accumulation of flavanones and flavones. Ma et al. (2021) found that MdERF109 promoted light-induced anthocyanin biosynthesis by directly binding to promoters of anthocyanin-related genes *MdCHS, MdUFGT*, and *MdbHLH3* in apple. Besides, An et al. (2020) showed that apple MdERF38 was able to promote the expression of anthocyanin biosynthetic genes *MdDFR*, *MdUF3GT*, *MdCHI* and *MdCHS* under drought stress. Cao et al. (2021) suggested that four lily (*Lilium brownii* var. *Viridulum*) transcription factors, ERF4, ERF WIN1-like, ERF061 and ERF071-like, might negatively regulate anthocyanin accumulation by directly modulating *LhMYBSPLATTER* gene. In short, a series of ERFs were confirmed to bind directly to promoters of genes involved in flavonoids synthesis and regulate their transcription under abiotic stresses. Current reports focus mainly on anthocyanidins synthesis in these processes, it is still not clear whether and how ERFs regulate the accumulation of flavonoids in other subclasses.

Comprehensively correlating subgroups and their biological functions, we conclude that the ERFs from 3a, 3d, 3e, 6b, 6e, 7a, 7d, 8b and 8c subgroup engage in drought, salinity, cold, heat, heavy metal and nutrition stress *via* binding to the GCC-box or/and DRE/CRT element of stress responsive genes. Notably, some of these subgroups could also induce flavonoid biosynthesis. We conjecture that ERF transcription factors in 8c subgroup are capable of binding to GCC-box or DRE/CRT element in drought, salinity, and cold and nutrition responsive genes to further modulate the synthesis of flavonoids, which regulates the tolerance of plant suffering corresponding stress. In like manner, ERF subgroup 6b are able to interact with GCC-box or DRE/CRT element in promoter of stress responsive genes to participate in flavonoid biosynthesis under drought, cold and salinity stress, while the 3a subgroup relates to drought and cold stress. Similarly, ERFs members of subgroup 6e and 7d tends to recognize GCC-box or DRE/CRT element of salinity-responsive genes to change the biosynthesis of flavonoids, when 6e and 7d are associated with drought and cold stress, respectively. Meanwhile, it also makes sense that ERFs in subgroup 3c regulate the synthesis of flavonoids in response to drought, salinity and cold stress. Nevertheless, our conjecture still needs further confirmation to reveal the molecular mechanism during the process.

## Conclusion

In this review, a comprehensive analysis of the ERF subfamily regarding the phylogenetic relationships, conserved motifs, *cis*-acting elements, stress response and regulation mechanism of ERF transcriptional activity was performed. ERFs are plant specific transcription factors, which play an important role in abiotic stresses, such as drought, salinity, chilling and some other adversities. Under these stresses, A series of post-translational modifications such as phosphorylation and ubiquitination affect the transcriptional activity of ERFs. ERFs are activated by mitogen-activated protein kinase induced phosphorylation, forming stable complexes with other transcriptional regulators and structural protein, then binding to *cis*-element in promoter regions of stress responsive genes. Generally, most ERFs were reported to bind specifically to the GCC cis-element, while the DREBs recognizes DRE/CRT cis-element to confer resistance to abiotic stresses. Beyond that, ERFs also modulate the synthesis of diverse metabolites, including proline, malondialdehyde and flavonoids etc. Act as an antioxidative agent, flavonoids are capable of scavenging ROS generated in plants during abiotic stresses.

ERF is a critical downstream component of the ethylene signaling pathway. Though previous transcriptome results suggested that large numbers of ERF genes of different subgroups play varied roles in response to abiotic stresses, very few interact proteins and target genes of them have been comprehensively annotated, and the molecular mechanism how stress signals been transited to ERFs and how ERFs regulate the transcriptional expression of stress responsive genes remains poorly understood and need further exploration.

## Author contributions

YW, XL, JZ, HZ, ST, WX, JP, and FY analyzed the phylogenetic relationships of ERF family members. EP conceived the original idea for the review. All authors wrote the manuscript. All authors contributed to the article and approved the submitted version.

## Funding

This work was supported by grants from the National Science Foundation of China (grants no. 31970286 and 31301053 to EP), the Natural Science Foundation of Zhejiang Province (grants no. LY22C020001 and LY17C020004 to EP) and the Hangzhou Science and Technology Bureau (grant no. 20170432B01 to EP).

## Conflict of interest

The authors declare that the research was conducted in the absence of any commercial or financial relationships that could be construed as a potential conflict of interest.

## Publisher’s note

All claims expressed in this article are solely those of the authors and do not necessarily represent those of their affiliated organizations, or those of the publisher, the editors and the reviewers. Any product that may be evaluated in this article, or claim that may be made by its manufacturer, is not guaranteed or endorsed by the publisher.
