# Supplementary material for: ERF subfamily transcription factors and their function in plant responses to abiotic stresses
Source: Front Plant Sci. 2022 Nov 30;13:1042084. doi: 10.3389/fpls.2022.1042084 (PMC9748296; doi:10.3389/fpls.2022.1042084)

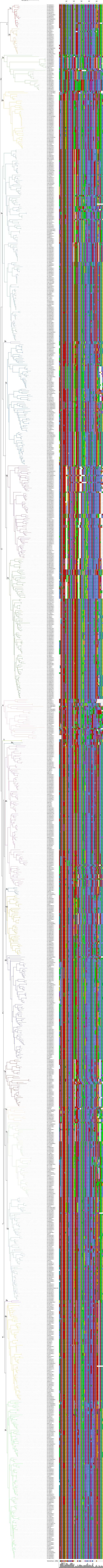

Supplement: Supplementary file 1 [file Image_1.pdf]
